# Sustainable, solvent-free exfoliation of 2D materials for thermally conductive metal powder coatings

**DOI:** 10.1038/s41699-026-00680-7

**Published:** 2026-02-18

**Authors:** Apostolos Koutsioukis, Siyuan Ruan, Ruben Cabello, Hyunjong Lee, Ilias Μ. Oikonomou, Xuyun Guo, Arnoldas Sasnauskas, José M. Munuera, Aran Rafferty, Yifeng Xiong, Sergi Dosta Parras, Wessel W. Wits, Shuo Yin, Jonathan Coleman, Rocco Lupoi, Valeria Nicolosi

**Affiliations:** 1 School of Chemistry, CRANN and AMBER Research Centres, Trinity College Dublin, Dublin, Ireland; 2 Department of Mechanical Manufacturing & Biomedical Engineering, Trinity College Dublin, The University of Dublin, Dublin, Ireland; 3 SDT, Departament de Ciència de Materials i Química Física, Universitat de Barcelona, Barcelona, Spain; 4 Faculty of Electrical Engineering, Mathematics and Computer Science, University of Twente, Enschede, The Netherlands; 5 School of Physics, CRANN & AMBER Research Centres, Trinity College Dublin, Dublin 2, Ireland; 6 Instituto de Ciencia y Tecnología del Carbono, INCAR-CSIC, Oviedo, Spain; 7 Centre of Excellence for Advanced Materials (CEAM), Dongguan, China; 8 NLR—Royal Netherlands Aerospace Centre, Marknesse, The Netherlands

**Keywords:** Chemistry, Materials science, Nanoscience and technology

## Abstract

2D nanomaterials offer unique functional properties when combined with metal powder feedstock, enabling advanced composites for engineering and energy applications^1^. Scalable fabrication of nanosheet-reinforced metal matrix composites (**2D**MMCs) remains challenging. In this study, we first exfoliate 2D materials using a solvent-free ball milling approach, using graphene and hexagonal boron nitride (hBN) as demonstrators, and then attach the resulting 2D nanoplatelets onto a wide range of metal powders, including copper (Cu), titanium (Ti-6Al-4V), aluminum (AlSi10Mg), and stainless steel (SS316L). To provide a mechanistic understanding of exfoliation, we use density functional theory (DFT) and discrete element method (DEM) simulations, offering new insights into the forces that drive nanosheet exfoliation. The resulting **2D**MMC powders combine excellent scalability and effectiveness. After consolidation, titanium alloy/graphene systems reaching thermal conductivity values of 17 W·m⁻¹·K⁻¹, comparable or superior to previous reports. Finally, we showcase their printability, confirming compatibility with large-scale manufacturing techniques and highlighting their potential for next-generation thermal applications.

## Introduction

Over the past decade, 2D materials have emerged as pivotal elements of the nanotechnology revolution due to their exceptional chemical, mechanical, and electrical properties arising directly from their atomic structure^2–5^. Their ultrathin nature allows atomic-scale control of properties, making them attractive in various technological fields. Each 2D material offers distinct advantages: graphene is an outstanding thermal and electrical conductor; hexagonal boron nitride (hBN) is an electrical insulator with excellent thermal conductivity; transition metal dichalcogenides (TMDs) such as MoS₂ and WS₂ serve as semiconductors for electronic and optoelectronic devices. The growing family of layered materials continues to expand opportunities across electronics, energy storage, and thermal management^3,6^.

Extensive research on 2D materials has led to a plethora of various approaches for producing monolayers and a few layers of 2D nanoplatelets including liquid phase exfoliation^7,8^, intercalation^2^, and top-down mechanical milling of 2D crystals^9^. Among these, mechanical ball milling stands out for its scalability and simplicity. However, it still faces important limitations.

Traditionally, the ball milling requires long processing times^10–12^ and the use of additives such as solvents (e.g., DMF^13^), polymers (e.g., melamine^14^), or viscous media^15–18^. While viscous media can localize shear forces and enhance the exfoliation efficiency, they often involve complex multi-step procedures and introduce impurities that compromise the structural integrity and integration potential of the nanoplatelets. As a result, the full potential of ball milling as a scalable and sustainable technique remains underexploited. On this, we redefine the method. By optimizing energy transfer and milling dynamics, we achieve efficient exfoliation and direct coating of metal powders without any external additives^18–20^.

Metals in parallel have a crucial role in the human progress from Bronze Age to today driving innovation and serving as vital components for society^21^. Their durability under extreme conditions makes them indispensable in fields such as energy, transportation, healthcare, and infrastructure^22^. In additive manufacturing (AM) especially they provide high functionality and excellent formability, enabling the design of complex, lightweight, and robust components. Studies on high-entropy alloys (HEAs)^23^, which combine multiple elements in equal proportions, further highlight the potential to create materials with tailored properties for next-generation applications. Together with their recyclability, metals are a game changer for sustainable technologies, addressing pressing issues such as environmental pollution and resource depletion^21^.

Combining 2D materials capabilities with metals allows the creation of composites with new properties that are otherwise impossible to achieve, unlocking advancements beyond the current limits. For example, integrating graphene or hBN into copper or titanium alloys could transform heat—dissipation strategies—a critical challenge faced by the electronics industry today^23^. However, common ways to prepare such composites (molten metallurgy, powder metallurgy, or electrodeposition) face major challenges. These challenges include, low scalability, inadequate wettability and undesirable interfacial reactions^22–26^ between the 2D materials with the metal powders.

This study presents a sustainable, gram-scale strategy for coating metal powders with 2D materials. Our method starts with solvent-free ball milling exfoliation of graphite and hBN. Using density functional theory (DFT) and discrete element method (DEM) simulations, we establish a mechanistic understanding of the process. This enables uniform coverage across a wide range of metal powders, including copper, aluminum, titanium alloys, and stainless steel. The resulting **2D**MMC powders display excellent thermal performance, particularly in titanium–graphene systems, and are fully compatible with additive manufacturing. After consolidation via Laser Powder Bed Fusion (LPBF), the titanium–graphene systems maintain their superior thermal properties. Overall, this approach provides a direct pathway from nanoscale engineering to scalable, real-world thermal solutions.

## Results

### Solvent-free exfoliation of 2D materials

To achieve uniform and defect-free coatings of metal powders with 2D materials, we developed a two-step solvent-free ball-milling strategy. In the first step, graphite crystals are exfoliated into high-quality graphene nanoplatelets while preserving their structural integrity and mechanical flexibility. In the second step, these nanoplatelets are used to coat a variety of metal powders through a subsequent ball milling process, enabling effective attachment of the 2D phase. This solvent-free approach addresses both the challenges of producing high-quality 2D nanoplatelets and achieving uniform coverage of various metal powders without the use of solvents.

**Fig. 1:**
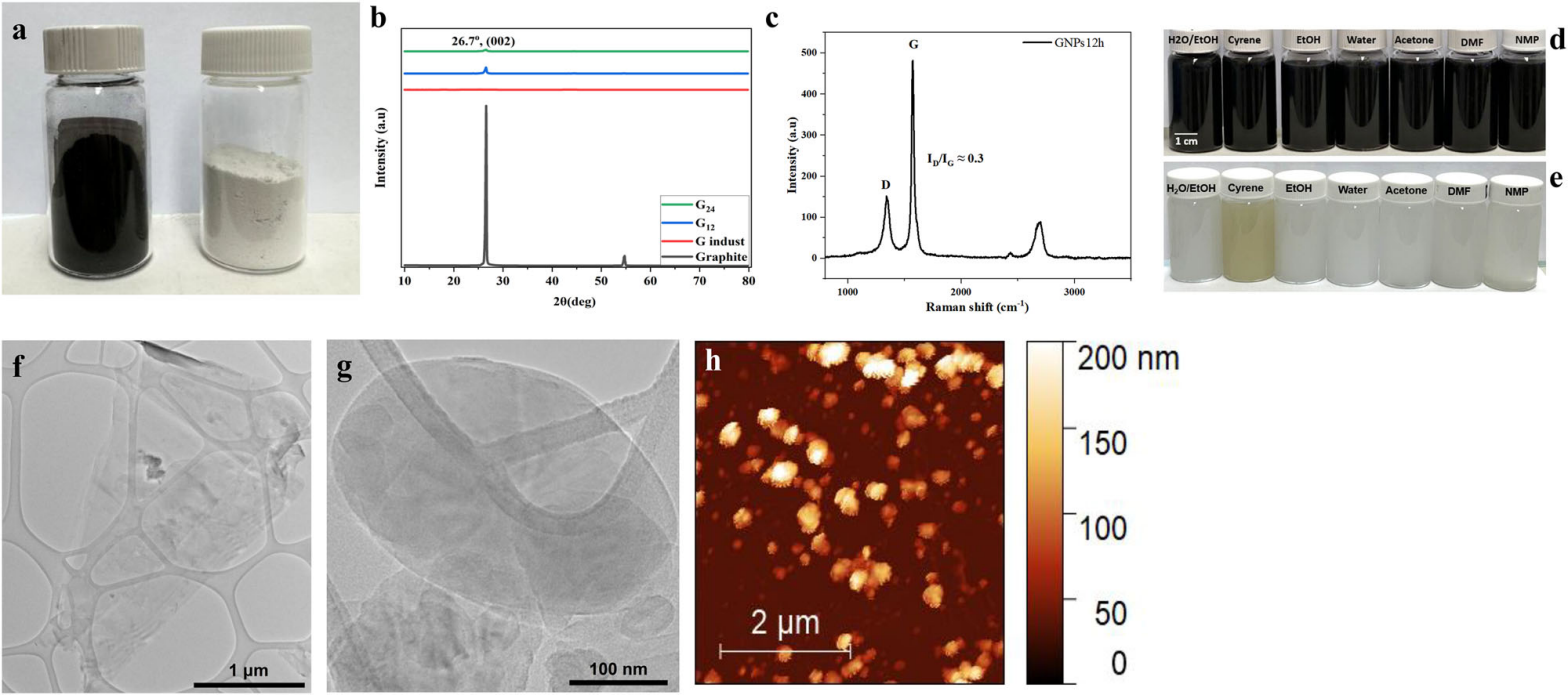
Physical and spectroscopic characterization of 2D nanoplatelets—exploring the dry ball milling technique beyond Graphene. **a** Photograph of as-produced graphene nanoplatelets (left) and the boron nitride nanoplatelets; **b** X-ray powder diffraction (XRD) patterns of pristine graphite, industrial graphene and graphene nanoplatelets after 12 and 24 h of ball milling; **c** Raman spectra of graphene nanoplatelets at 12 h, showing the characteristic D and G bands with a low I_D_ /I_G_ ratio; **d**, **e** Dispersibility of graphene and hBN nanoplatelets at 12 h in organic solvents, water and sustainable cyrene **f** Transmittion electron microscopy (TEM) image of graphene nanoplatelets at 12 h; **g** TEM image of hBN nanoplatelets at 12 h; **h** AFM image of hBN nanoplatelets at 12 h.

We ball-milled graphite powder for durations ranging from 2 to 48 h, using a constant ball-to-powder ratio and constant speed to control the energy input (Fig. 1a, Fig. S1). Duration of ball milling of the graphite up to 4 h results in poor dispersibility in isopropanol (IPA), whereas after 6 h, the material disperses readily in IPA, indicating the onset of exfoliation (Fig. 1d, Fig. S1a, Fig. S1d). XRD analysis of the samples support this trend, with a distinct (002) carbon peak at 26.7° emerging clearly at 6, 12, and 24 h, corresponding to highly ordered stacking of graphene nanoplatelets (Fig. 1b, Fig. S1e). A significant reduction in peak intensity at 12 h suggests substantial exfoliation and thinning into graphene nanoplatelets, while the persistence of the (002) peak indicates that the lattice remains ordered. TEM images further demonstrate the production high quality thin graphene nanoplatelets, with improved dispersibility across a range of solvents (Fig. 1f). Raman analysis and FWHM measurements reveal an *I*_D_/*I*_G_ ratio to 0.3 (Fig. 1c), indicating low defect density and well-ordered graphene layer (Fig. S1b). Thermogravimetric analysis shows, in the case of 12 h, that the graphene nanoplatelets exhibit typical thermal behavior of graphene, with only a slight weight loss observed above 800 °C compared to graphite (Fig. S1c).

Laser diffraction particle size analysis shows a reduction in mean particle diameter D^3,4^ as a function of ball-milling time, i.e., from 349 μm for graphite ball-milled for 4 h at 500 rpm, to 208 μm after 12 h, to 54 μm after 48 h (Table S1). Ball milling was also found to result in an increase in specific surface area from 1.3 m² g⁻¹ for un-ball-milled graphite to values approaching 400 m² g⁻¹ for samples milled for 48 h at 500 rpm. To assess the suitability of exfoliated nanoplatelets for metal surface attachment, we examine how morphology and surface are evolving with ball milling time. Graphite exhibited the lowest aspect ratio values, consistent with its intrinsic flake-like morphology. Upon increasing the milling duration, this morphological evolution correlated with higher surface area, indicating the shape regularity enhances the accessible surface. Notably, while the average particle diameter remained relatively constant, prolonged milling led to the formation of more spherical particles.

To understand the mechanism behind solvent-free exfoliation of graphite, we focus on identifying critical stages of the exfoliation process without the use of polymers or high-viscosity solvents. Our results showcase that the fragmentation of graphite plays a dual role: it increases the surface area while lowering the energy barrier for exfoliation. A combined experimental and simulation approach—including Density Functional Theory (DFT) calculations of exfoliation energy and Discrete Element Method (DEM) modeling of ball-milling dynamics—shows that as particle size decreases under controlled milling speeds, the exfoliation process becomes more energy efficient. This indicates that solvent-free ball milling can achieve effective exfoliation by optimizing mechanical fragmentation and shear transfer to the target materials, offering a simpler and scalable route for graphene nanoplatelets production.

### Mechanism of solvent-free exfoliation of 2D materials

**Fig. 2:**
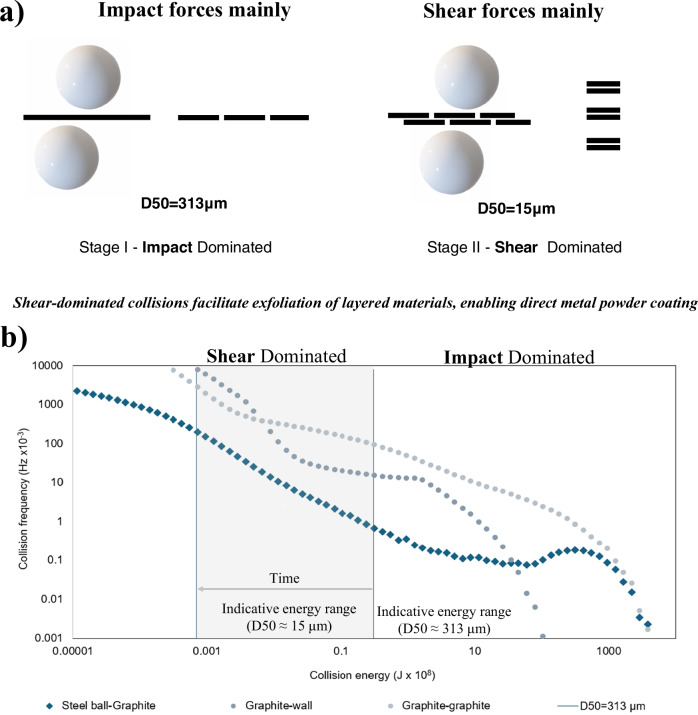
Mechanism of solvent-free ball milling exfoliation of 2D materials. **a** Schematic representation of the exfoliation mechanism during solvent-free ball milling. Early impact-dominated collisions mainly induce graphite fragmentation (D50 ≈ 313 μm), while continued milling shifts the regime toward shear-dominated interactions that promote layer exfoliation into thinner 2D nanoplatelets (D50 ≈ 15 μm); **b** DEM-simulated collision energy–frequency distributions at 500 rpm, showing an increasing contribution of shear-dominated collisions above the exfoliation threshold as particle size decreases.

DFT calculations confirm that exfoliating graphene layers requires ~0.4 J/m² ^27,28^. In our approach, we reduce the graphite size from D50 = 313 μm to 15 μm at 500 rpm. This reduction creates a regime where exfoliation energy was significantly lowered. By reducing the particle size, the collisions occur with an energy that is better suited for exfoliation. As a result, the overall exfoliation energy is significantly reduced. At 15 μm, only ~7 × 10⁻¹¹ J per shear was required, with ~8 × 10⁷ such events occurring every second. In contrast, the starting graphite at 313 μm experienced only ~1.5 × 10⁴ effective collisions per second. This dramatic shift highlights how controlled fragmentation increases collision frequency and reduces the exfoliation energy barrier. At lower speeds (e.g., 150 rpm), both fragmentation and exfoliation are both delayed. At 15 μm, the energy required for each shear significantly slower reduction in particle size and a longer period needed to initiate exfoliation (Fig. 2a).

DEM simulations further support our findings by showing that, at 500 rpm the collision energies generated from shear interactions are sufficient to promote exfoliation of the graphite crystals. According to (Fig. 2b), the number of the collision above the exfoliation threshold increased steadily as particle size decreased. Together, these results demonstrate a sustainable, scalable route to exfoliate 2D materials, suitable to coat metal powders^29^. The DEM simulations adopt a simplified framework and do not explicitly account for van der Waals interactions or possible re-agglomeration of exfoliated nanoplatelets, which constitutes a known limitation of the model. Nevertheless, the simulations capture the dominant collision regimes governing exfoliation and are consistent with the experimentally observed behaviors.

To test the versatility of our approach, we applied the same parameters from the two-step strategy to hBN (Fig. 1e, Fig. S2)^30–33^. TEM analysis confirmed the layered structure (Fig. 1g), while XRD showed a peak at 26.96° (002) in both bulk hBN and the exfoliated nanoplatelets, indicating that the crystal structure is preserved (Fig. S2a, S2c, d). Raman spectra exhibited a peak at 1369 cm⁻¹, slightly shifted compared to unexfoliated hBN, further supporting successful exfoliation (Fig. S2b). Finally, AFM analysis of ball-milled hBN nanoplatelets revealed an average thickness of about 13 layers, demonstrating the robustness of our solvent-free milling approach (Fig. 1h).

According to the previous studies, as the thickness of graphite or hBN decreases, the nanoplatelets show enhanced mechanical compliance and surface energy^34,35^. In this direction, our method enables the uniform coating of a broad range of metal powders, including copper, titanium and aluminum alloys, as well as stainless steels^32–36^.

### Attachment of 2D nanoplatelets on metal particles (2DMMCs)

**Fig. 3:**
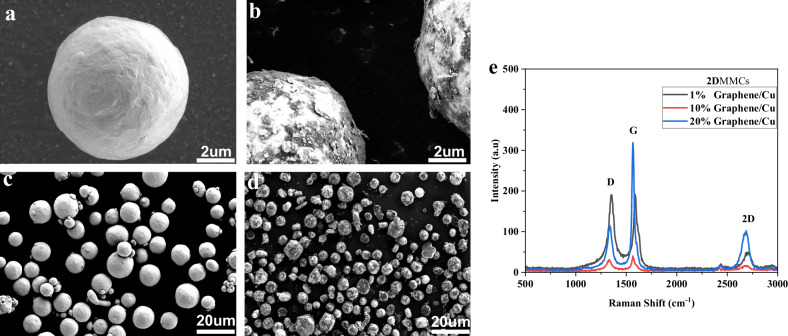
Graphene coating of Cu metal particle. **a** SEM images of **a**, **c** pure copper particle; **b**, **d** Graphene/Cu **2D**MMC composite and **e** Raman spectra of Graphene/Cu **2D**MMCs at different concentrations 1 wt%, 10 wt%, 20 wt%.

For the attachment of 2D nanoplatelets on the metal surfaces, we follow a mild solvent-free approach (150 rpm, 6 h) starting with copper particles and varying graphene nanoplatelets loadings (from 1 wt% to 20 wt%). SEM images of Cu/graphene **2D**MMCs reveal successful surface coverage of graphene even at low content (Fig. 3a-d). Raman spectroscopy further identifies characteristic graphene peaks, providing insights into the successful of coverage of the metal particle (Fig. 3e). EBSD on the pure copper before and after ball milling shows grain refinement without compromising the powder’s suitability. Misorentation mapping further indicates low and stable internal stress levels, validating the mild milling conditions for effective metal surface coverage^37^(Fig. S3).

**Fig. 4:**
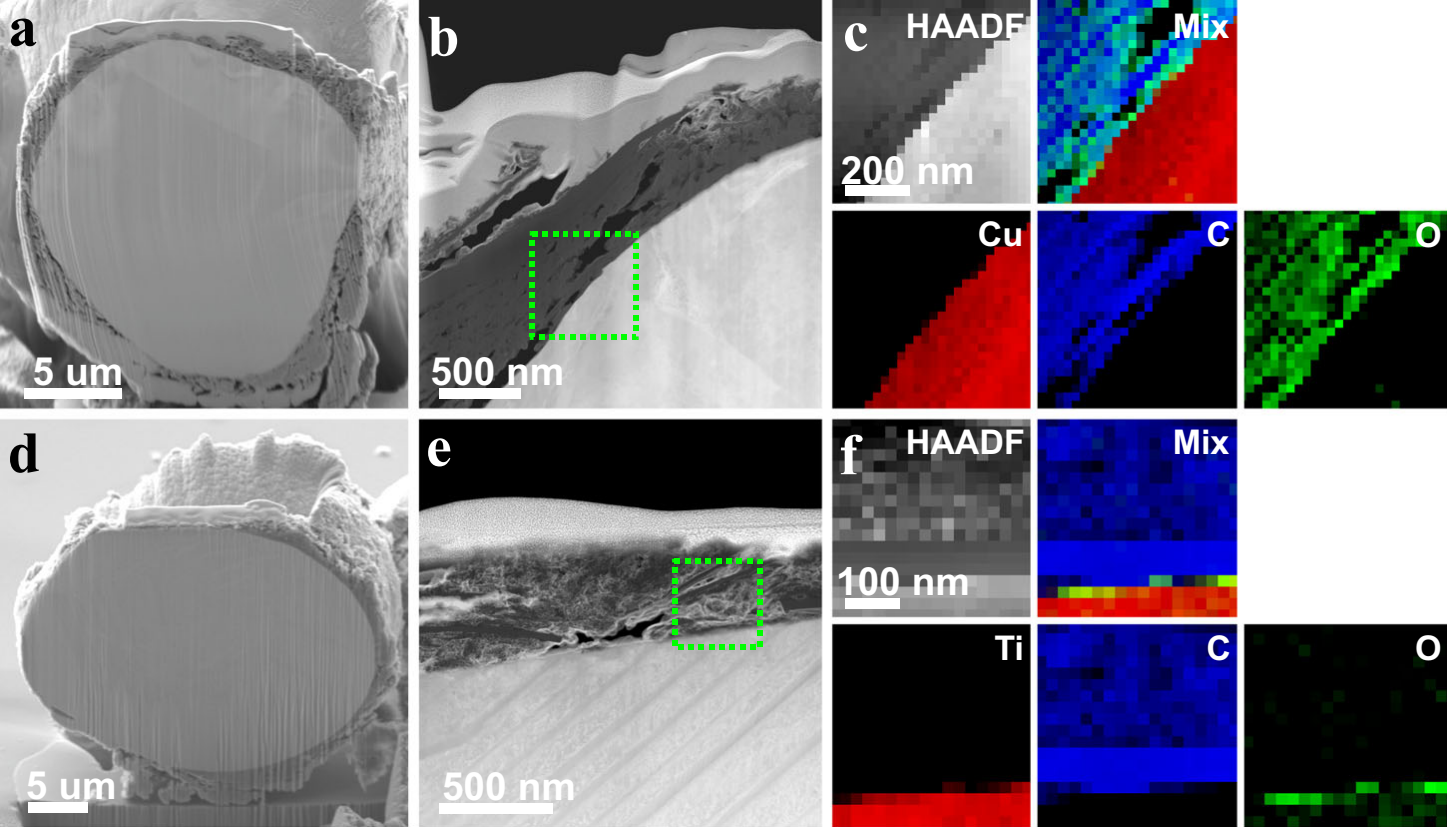
Cross-sectional characterization of graphene-coated metal powders (2DMMCs). **a-c Graphene/Cu 2D**MMC; **a** cross-sectional SEM image of a single Cu particle **b** HAADF-STEM image highlighting continuous graphene nanoplatelets surface layer (green dashed box), and **c** HAADF-STEM image and corresponding EELS maps of Graphene/Cu **2D**MMC **d-f Graphene/Titanium alloy 2D**MMC; **d** cross-sectional SEM image of a Ti alloy particle, **e** HAADF-STEM image showing graphene nanoplatelets-containing surface layer (green dashed box), and **f** HAADF-STEM image and corresponding EELS maps of Graphene/Titanium alloy **2D**MMC.

Cross-section analysis was performed on graphene/copper and graphene/titanium-alloy **2D**MMCs composites to investigate the graphene distribution on the metal particle surface. (Fig. 4a-c) show a representative HAADF-STEM image of a graphene/copper composite particle; the overlay in (Fig. 4c) showcases the corresponding energy loss spectroscopy (EELS) maps, showcasing the presence of copper (red), carbon (blue), and oxygen (green) layers, and confirming the uniform distribution of graphene around the copper particle. The same type of characterization follows on graphene/titanium alloy composites (Fig. 4d-f), confirming the uniform distribution of graphene around the titanium alloy particle. Both material systems exhibit smooth and continuous interfaces without significant voids or gaps, indicating robust interfacial mechanical bonding between the graphene nanoplatelets and the metal powder surface. Cross-sectional characterization of copper and titanium alloy **2D**MMCs using TEM, HRTEM and SAED, further confirms the presence of a graphene layer on the metal surface for both titanium alloy and copper particles (Fig. S4-S5). Based on this mechanical bonding^38,39^, primarily driven by van der Waals interactions between the graphene (2D material) and metal particle, we demonstrate further that the thickness of the coating layer on the metal particles can be tailored and significantly increased from the nanoscale (120–320 nm) to the microscale (~1.2–1.8 μm), as shown in Fig. S6.

### Scalability and thermal performance of 2DMMCs

**Fig. 5:**
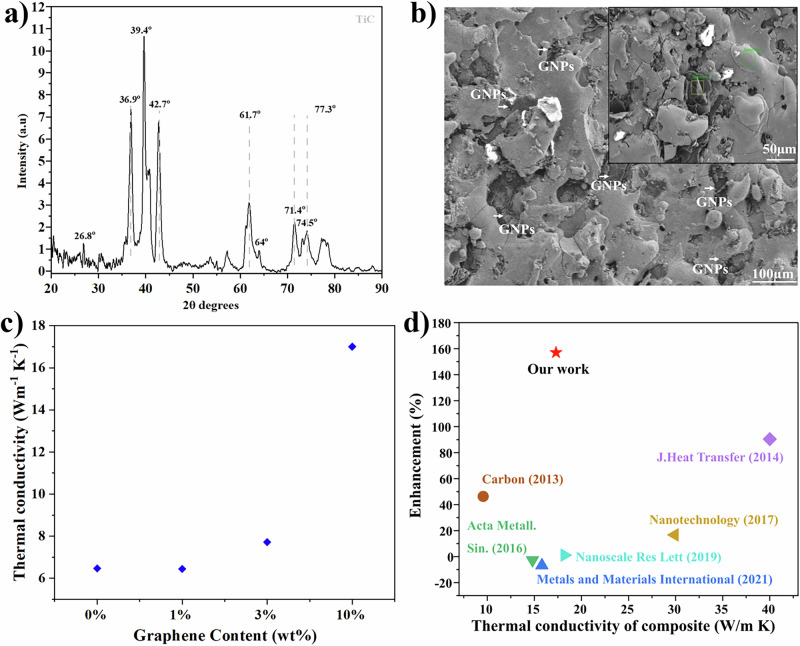
Thermal performance of 2DMMCs. **a** XRD pattern of the Ti alloy reinforced with 10 wt% graphene. XRD pattern of the consolidated Ti-graphene composite showing the characteristic TiC peak, confirming interfacial reactions during sintering. The pattern is lightly smoothed using a Savitzky–Golay filter (20 points) for clarity **b** SEM micrograph revealing a homogeneous distribution of graphene nanoplatelets within the titanium alloy matrix (inset: higher-magnification image highlighting embedded GNPs) **c** Thermal conductivity enhancement of Graphene/Ti alloy **2D**MMCs as a function of graphene content (wt%) **d** Comparison of thermal conductivity enhancement versus thermal conductivity of composite materials reported in the literature, highlighting the performance of the present work (red star).

After confirming the coverage of copper and titanium particles with graphene nanoplatelets, we extend our approach to a broad range of metal powders from stainless steel (SS316L), Ti64Al4V and AlSi10Mg alloys with both graphene and hBN. SEM images confirm seamless attachment of 2D nanoplatelets across all metals, showing robust and uniform coverage of the 2D phase. Further, this strategy on **2D**MMCs shows great potential for scalability: we demonstrate this by increasing graphene production in our small-scale laboratory starting from 8 to 24 g of graphene in 24 h, with similar results for hBN nanoplatelets (Fig. S7). Similarly, we can upscale the production of the **2D**MMCs composites from 1 g after 6 h of solvent-free ball milling to 480 g after 24 h following the same ball-to-powder ratio of small-scale production (Fig. S8). Beyond the industrial scale potential of our approach, the consolidated titanium alloy/graphene composites at this level exhibit significant thermal enhancements (Fig. 5).

Graphene-coated titanium alloy powders were consolidated using spark plasma sintering (SPS), resulting in dense and uniform composites. The conformal graphene coverage prior to sintering ensured homogeneous carbon distribution, which is critical for the formation of efficient thermal transport pathways^40,41^. At 10 wt% graphene, the composites reached a thermal conductivity of 17 W·m⁻¹·K⁻¹, approximately 2.5 times higher than that of uncoated Ti-6Al-4V (6.65 W·m⁻¹·K⁻¹) (Table S2). XRD analysis indicates the formation of strong Ti-C interfacial interactions, evidenced by reflections in the 36–42° range (Fig. 5a), which are consistent with the formation of carbide-like bonding at the graphene–titanium alloy interface during SPS processing. Given that the intrinsic thermal conductivity of Ti-6Al-4V(Fig. 5b), is approximately 6.65 W·m⁻¹·K⁻¹, the significantly higher value obtained here indicates an additional bulk contribution arising from the incorporation of graphene nanoplatelets and the formation of thermally conductive networks within the matrix (Fig. 5c). To the best of our knowledge^42–49^, the thermal conductivity values reported here rank among the highest for graphene/titanium composites produced by scalable methods (Fig. 5d), confirming the effectiveness of graphene incorporation and Ti-C interfacial interactions in enhancing thermal transport (Table S3).

### LPBF printability of 2DMMCs for additive manufacturing

Finally, to integrate our **2D**MMCs platform with large-scale manufacturing techniques, we demonstrate their printability using the LPBF technique. LPBF is a powerful additive manufacturing technique, enabling the creation of freeform geometries, complex features, and tool-less fabrication, with high resolution and excellent surface quality. Starting from titanium alloy composites with graphene or hBN nanoplatelets, as well as stainless steel 316 L with graphene, we achieved successful deposition of the 2D phase through metal melting, even at low nanosheet loadings. Raman spectroscopy of the printed parts confirmed the retention of graphene characteristics, with an *I*_D_/*I*_G_ ratio of 0.34, close to the initial nanoplatelets (Fig. S9). The 2D coverage of the metal particles facilitates high-quality, uniform printed structures, according to RAMAN and SEM, which revealed the presence of carbon from the graphene nanoplatelets compared to pure titanium (Figs. S10, S11). While further studies are needed to expand the material scope, these results underscore the robustness and versatility of our solvent-free approach for producing thermally enhanced, high-performance, and printable **2D**MMCs.

## Discussion

We demonstrate a sustainable and scalable method to produce 2D metal matrix composites (**2D**MMCs) via solvent-free ball milling, coating copper, titanium (Ti-6Al-4V), aluminum (AlSi10Mg) alloys, and stainless steel (SS316L) powders with 2D nanoplatelets. This approach avoids so high viscous solvents or surfactants and is compatible with industrial-scale manufacturing. For the exfoliation of 2D materials we conclude with a mechanistic insight from DFT and DEM simulations reveals optimal energy transfer and particle collision dynamics that reduce the interlayer energy barrier. For 15 μm particles, only ~7 × 10⁻¹¹ J per shear is needed, with ~8 × 10⁷ events per second over a thousand times more than the ~1.5 × 10⁴ effective collisions at 313 μm. We scale up the **2D**MMCs platform, achieving titanium-based composites with significantly enhanced thermal conductivity (17 W m⁻¹ K⁻¹ at 10 wt% graphene), attributed to newly formed carbon–titanium bonds. These composites are also fully printable via the LPBF technique. Finally, while this study focused primarily on the preparation of **2D**MMCs for thermal management applications, preliminary observations suggest excellent promise in the energy field as well.

## Methods

### Materials

Graphite (Graphite, 332461 Sigma-Aldrich. Boron nitride (boron nitride, 2555476, Sigma-Aldrich), Copper powder (−38 + 15 μm, Safina), AlSi_10_Mg (PowderRange—Carpenter Additive, 20–63 μm), Ti64Al4V (Powder Range— Carpenter Additive, 15–53 μm), Steel 316 L (Powder Range – Carpenter Additive, 15–45 μm).

### Preparation of 2DMMCs

In a typical experiment, 1 g of graphite flakes was mixed in milling jars (two to four mostly) under an argon atmosphere (Ag) and milled at 500 rpm for 2-48 h, with a powder-to-balls ratio: 1 to 20 (steel balls, 0.5 mm diameter) and each of the ball-milled graphene nanoplatelets was collected as G_*x*_ (*x* = 2, 6, 12, 24, 48 h). We followed the same procedure for the preparation of for few layer hBN nanoplatelets using the best conditions from the graphene method, mentioning this product as hBN_*x*._ For the large-scale production of graphene, we used the same ration of balls to powder with a mass of starting graphite of 3 g for each jar. After the end of the process, the 2D phase is used for the preparation of **2D**MMCs. A further step of preparing **2D**MMCs is followed up, by mixing the graphene or hBN nanoplatelets with metal powder (different mass ratios), a ratio of powder to balls, 1 to 10, under argon atmosphere for 6 h at 150 rpm. This method can apply to different metal powder of copper, AlSi_10_Mg, steel 316 L, and Ti64Al4V. The large scalability of this method can be expanded for 30 g of the **2D**MMCs composites (two to four mostly) with the same ratio of the balls to powder 1 to 10, at 150 rpm 6 h (in total 120 g of preparation at 6 h).

### Characterization of 2DMMCs

Scanning electron microscopy (SEM) was conducted using the Zeiss ULTRA plus Gemini SEM microscope in high vacuum mode with an acceleration voltage of 5 kV, a working distance of 6 mm, and a 30 µm aperture located in the Advanced Microscopy Laboratory (AML) of TCD.

Transmission electron microscopy (TEM) was performed using uncorrected FEI Titan with Schottky field emission S-FEG source operated at 300 kV. TEM images were recorded using a Gatan UltraScan CCD camera and electron energy-loss spectroscopy (EELS) mapping was carried out with Quantum Gatan Imaging Filter (GIF) detector with energy dispersion of 0.5 eV per channel.

Ball milling machine PQ-N2 500 mL × 4 planetary 220 V PQN2.220–50 Hz. X-ray diffraction (XRD) measurements were performed using a Panalytical X’Pert Pro-diffractometer with a Cu Kα source (*λ* = 1.5406 Å). Symmetric scans run over a 2*θ* range of 10° to 75°, with a step size of 0.0084°.

Raman spectroscopy performed using a WITec Alpha 300 R with 532 and 633 nm excitation lasers with a spectral grating of 1800 lines/mm and a 100× microscope aim (0.95 N.A., spot size ∼0.3 μm). Spectra taken with a laser power of <300 μW in order to minimize sample heating.

Thermogravimetric analysis (TGA) was conducted from 50 to 900 °C with the temperature rising rate of 10 °C/min under continuous nitrogen flow (PerkinElmer TGA 8000).

Particle Size Distribution Malvern Mastersizer 2000 (Model APA2000) Malvern Instruments Ltd., UK.

### Thermal conductivity measurements graphene/Ti Aalloy **2D**MMCs

For the thermal characterization of SPS Graphene/Ti alloy **2D**MMCs, we utilized the transient plane source (TPS) technique to measure their thermal conductivity^50^. TPS methodology was selected as it enables characterization of samples with various geometries, including slabs, rods, and bulk disks.

Accordingly, a cylindrical slab geometry with a high aspect ratio of diameter to thickness was chosen to ensure the in-plane thermal conductivity measurements of **2D**MMCs, by geometrically confining the predominant radial-directed heat flow compared to axial direction^51^.

Specifically, slabs with a diameter of 30 mm and a thickness of 1 mm are prepared using a wire electrical discharge machining tool (CUT AM 500, GF Machining Solutions, Switzerland), equipped with a 0.2 mm thick pure Mo wire.

The reproducibility and reliability of measurements were secured by conducting three repeated tests for each set. Each set is composed of two slabs sandwiching a 6.4mm-sized double-sided sensor (MP-1 two-sided sensors, Thermtest, Canada), measuring a total of three slabs.

As a result, these testing method enables the measurement of more than half of the entire SPS cylinder (30 mm diameter and 5–6 mm thickness), representing the overall thermal properties of Ti alloy-graphene **2D**MMCs.

## Supplementary information

2DMMCprep_SupplementaryNPJ_Final

## Data Availability

All the data generated or analyzed during this study are included in this published article and its supplementary file.
