# Supplementary material for: Sustainable, solvent-free exfoliation of 2D materials for thermally conductive metal powder coatings
Source: NPJ 2D Mater Appl. 2026 Feb 18;10(1):41. doi: 10.1038/s41699-026-00680-7 (PMC13031118; doi:10.1038/s41699-026-00680-7)
Supplement: Supplementary file 1 — 2DMMCprep_SupplementaryNPJ_Final [file 41699_2026_680_MOESM1_ESM.pdf]

Supplementary information

Sustainable, Solvent-Free Exfoliation of 2D Materials for Thermally Conductive Metal Powder Coatings

Apostolos Koutsioukis, Siyuan Ruan, Ruben Cabello, Hyunjong Lee, Ilias Oikonomou, Xuyun Guo, Arnoldas Sasnauskas, José M Munuera, Aran Rafferty, Yifeng Xiong, Sergi Dosta Parras, Wessel W. Wits, Shuo Yin, Jonathan Coleman, Rocco Lupoi, Valeria Nicolosi

Corresponding Authors Valeria Nicolosi (NICOLOV@tcd.ie) and Apostolos Koutsioukis (koutsioa@tcd.ie)

Solvent Free Exfoliation of 2D Materials

During the dry ball milling process, the balls inside the containers generate kinetic energy that triggers both physical and chemical changes in the graphite crystals (**Figure S1**). After 2 hours of mechanical grinding, the graphite sample (G2) is not dispersible in isopropyl alcohol (IPA), while the sample milled for 6 hours (G6) shows good dispersibility in IPA. This indicates the exfoliation of the graphite crystals into graphene nanoplatelets (GNPs) . This observation suggests that during the initial 2 hours of ball milling, the mechanical grinding primarily reduces the size of the graphite crystals through impact energy. However, after 6 hours of milling (**Figure S1a)**, the process not only reduces the crystal size but also initiates exfoliation into graphene nanoplatelets due to the increased shear energy. Therefore, at 500 rpm with a powder-to-ball ratio of 1:20, impact energy primarily influences the initial stages of ball milling, resulting in the reduction of the graphite crystal size. As ball milling progresses beyond 6 hours, shear energy becomes increasingly influential, facilitating the effective exfoliation of the graphite crystal. During the period from 6 to 48 hours of ball milling, both in-plane and out-of-plane breakage of the graphite occurs simultaneously. The Gx samples with different ball-milling durations were compared with industrially graphene (Gelicarb) **(Figure S1a).** The Raman spectra of the G powders at different milling times are at 514 nm by applying the powder on a quartz slide. As it occurs, GNPs powders have three major bands. The D band at 1346 cm^{-1} corresponds to first-order phonons, indicating defects in the sp^2 hybridized carbon lattice. The G band at 1580 cm^{-1} relates to the Raman-allowed band, reflecting the original state of the sp^2 hybridized carbon lattice. The 2D band at 2688 cm^{-1} is due to second-order phonons and is an indicator of the number of graphene layers (**Figure S1b)**. The intensity ratios of the D and G bands imply the presence of oxygen functional groups or broken aromatic rings. The I_{2D}/I_G ratios, ranging from 0.34 for G_{12} to 0.55 for G_{24}, suggest the existence of few layers in the spotted graphene nanoplatelet accordance to with TEM images. Notably, this ratio is lower than those of graphene oxide and reduced graphene oxide, indicating well-preserved high

quality for the preparation of GNPs. Another indicator of the structural quality of our samples is the FWHM measurement from Raman spectroscopy. The FWHM of the GNPs samples from 12 to 48 hours range from 70 cm^{-1} to 80 cm^{-1}, implying a layered graphene structure. The TGA results highlight significant variations in the weight percentage of oxides on the graphene layers, with G12 exhibiting a low oxide content of approximately 5 wt.%, while G48 shows a substantial increase to 44 wt.%, indicating that longer ball milling times lead to more pronounced oxidation (**Figure S1c**).The GNPs via dry ball milling exhibit the characteristic UV-Vis at 268nm, due to carbon bonds both for 12h and 24h (**Figure S1d**).

The XRD patterns (**Figure S1e**) reveal that the peak at 26.7° (002) indicates the highly ordered stacking of graphene layers. The crystallite size decreases from 0.34 nm in graphite to 10.94 nm in G_{24}, compared to 2 nm in industrial graphene (Gelicarb), confirming that prolonged ball milling results in thinner graphene layers and larger graphene domains. Additionally, the intensity ratio of the (002) to (100) peaks decreases, suggesting a reduction in graphite thickness. The crystalline structure of graphite is preserved, as indicated by the broad peaks at 43-45° (100)/(101) and 76° (110), corresponding to the 2D in-plane symmetry along the graphene sheet. A weak (002) peak at 26° in all samples suggests parallel graphene layers likely due to nanosheet restacking. These findings demonstrate the efficacy of ball milling in exfoliating graphite into high-quality GNPs without additives nor solvents while maintaining the crystalline structure of graphene **(Figure S1)** .

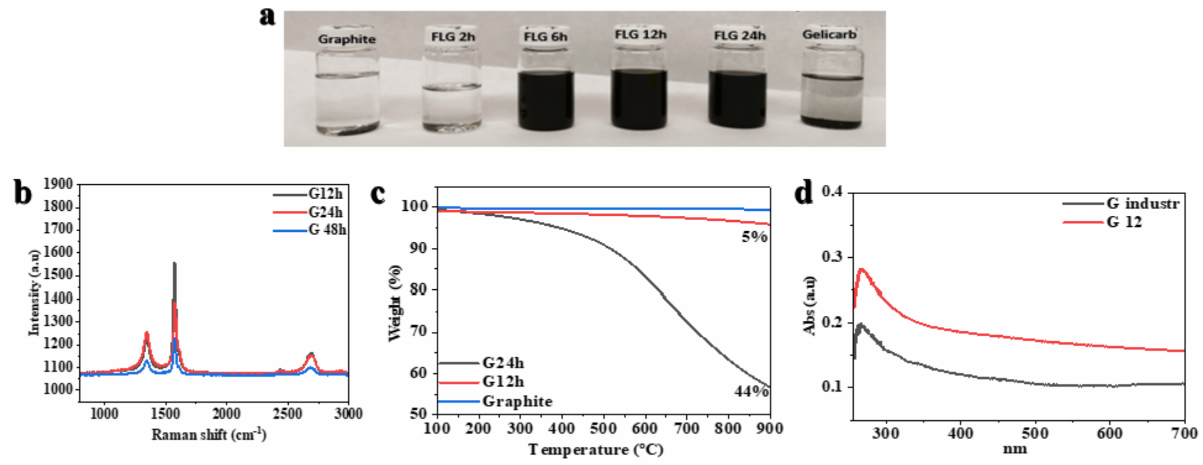

e

Sample	Intensity (a.u)	2θ (degree)	Size of crystallite (nm)	Intensity ratio of (002)/ (100)
Graphite	20003042	26.69	0.34	-
G12	77351	26.51	20.14	**38.91**
G24	20709	26.51	10.94	**20.71**
Graphene Industrial	1963	25.49	2	-

Supplementary Figure S1. Physical and spectroscopic properties of graphene nanoplatelets (GNPs) prepared by dry ball milling **a** Dispersibility comparison of GNPs at different milling durations (2h, 6h, 12h, 24h) versus industrial graphene and starting graphite powder **b** Raman spectra of GNPs at 12h, 24h, and 48h **c** TGA curves comparing GNPs at different rpm with starting graphite **d** UV-Vis absorption spectra of GNPs at 12h compared to industrial graphene **e** XRD peaks of GNPs at different hours (G12, G24) compared with graphene industrial.

The universality of this technique is further demonstrated with hexagonal boron nitride, another remarkable 2D material, confirming its reproducibility on large scale following the successful preparation of graphene nanoplatelets through dry ball milling techniques. We use the same parameters of 12h ball milling process for the hexagonal boron nitride (hBN), with a powder-to-ball ratio of 1:20 under an argon atmosphere similar to the graphene nanoplatelets. According to XRD **(Figure S2a)**, the hBN nanoplatelets have well crystallized structures. Both bulk hBN and hBN nanoplatelets exhibit a characteristic peak at 26.96° (002), confirming the preservation of the BN phase structure without significant damage during exfoliation. The slight shift to a lower angle indicates increased layer spacing due to the exfoliation process. Peaks at 26.6°, 41.6°, 43.1°, 54.9°, and 75.8° correspond to the (002), (100), (101), (004), and (110) planes of hBN. The sharp intense peak observed at $2\theta = 26.5°$ confirms the formation of hBN with the (002) plane **(Figure S2a)**. The absence of residual peaks across the XRD spectrum confirms the purity of the synthesized hBN nanoplatelets . We compare the Raman spectra of hBN produced via dry ball milling for 12 hours with that of hBN obtained through liquid phase exfoliation in IPA **(Figure S2b)**. The results showcase the efficiency of our dry ball milling method, with a peak at 1371 cm^{-1} for liquid phase exfoliation (corresponding to the E2g mode) and a slight downshift to 1369 cm^{-1} for dry ball milling according to Ghosh. This downshift can be attributed to lattice thermal expansion or softening of BN bonds during the ball milling process. Finally, TEM characterization of hBN (**Figure 1e**) leads from BN crystals into rounded flakes with a lateral size of 250nm.Thermal stability of Hbn represented at (**Figure 2c).** UV-Vis spectra reveal sharp absorption peak at 210 nm The optical band gap of boron nitride is approximately 5.12 eV, calculated using a Tauc plot, which is very close to the theoretical band gap value of 5.5 eV **(Figure S2d)**.

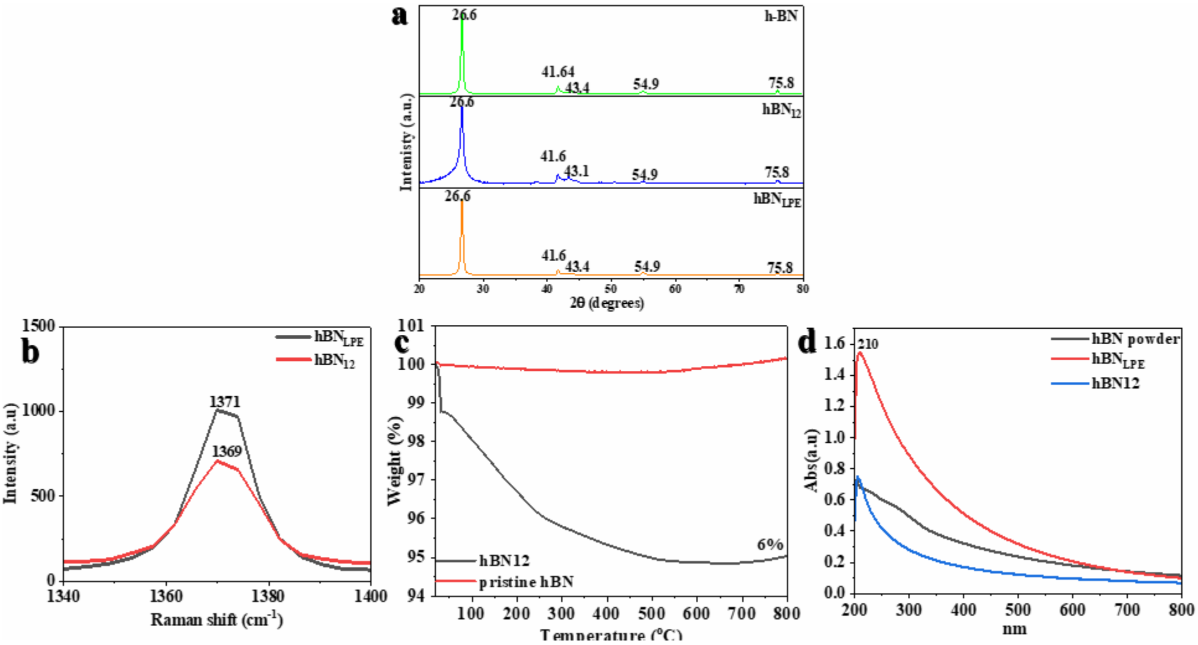

Supplementary Figure S2. Physical and spectroscopic properties of hBN nanoplatelets prepared by dry ball milling **a** XRD comparison of hBN nanoplatelets to hBN powder and LPE-hBN (liquid phase exfoliation in IPA of hBN powder) **b** Raman spectra comparison of hBN nanoplatelets at 12h and LPE-hBN **c** TGA curves comparing hBN at 12h and hBN powder starting powder nanoplatelets **d** UV-Vis absorption spectra of ball-milled hBN, LPE-hBN, and pristine hBN.

Mechanism of solvent-free exfoliation of 2D materials

Supplementary Table S1. Experimental characterization of exfoliated graphite samples, Raman spectroscopy data, XRD patterns and laser diffraction-based particle size diameter, used as input parameters for the DFT and DEM modelling of exfoliation dynamics and upscaling results of graphene exfoliation.

Sample	Milling speed (rpm)	Time (h)	D [4,3] µm	Raman (I_D/I_G)	Raman (I_{2D}/I_G)	XRD (2θ°)/Intensity (a.u.)
**1**	150	2	598	0.0488	0.2000	26.61 / 15793
**2**	300		593	0.0833	0.1770	
**3**	400		459	0.1406	0.2153	
**4**	500	4	349	0.0236	0.2017	26.62 / 15485
**5**	150		602	0.0907	0.2060	26.68/

**6**	300	6	605	0.0573	0.2445	15.964
**7**	400		464	0.0725	0.2178	
**8**	500	8	207	0.0849	0.2116	26.44 /14438
**9**	500	10	257	0.0539	0.2175	26.31 / 13771
10	150		647	0.0274	0.2588	26.5/ 15964
11	300		644	0.0544	0.1924	26.5/ 15964 (for300rpm)
12	400	12	445	0.0952	0.2098	26.69 / 13448 (for 400rpm)
13	500		208	0.3129	0.1901	26.36/ 1255 (for 500rpm)
**14**	500	18	123	0.0440	0.2270	26.60 / 6226
**15**	400	24	355	0.87	0.42	26.35/13006<br>26.52/14591
**16**	400	36	260	0.41	0.58	26.58/16000
**17**	300		530	0.0820	0.2753	26.51/15941
**18**	400	48	242	0.0213	0.2238	26.51/ 11.736
**19**	500		54	0.1503	0.1785	26.51/1839

Attachment of 2D nanoplatelets on metal particles (2DMMCs)

The EBSD maps in **Figure S3** illustrate the distribution of grains in both untreated and ball-milled powder samples of the copper powder at 150rpm for 6h which is our demonstrator. The untreated powder exhibits an average grain size of 4.79 ±4.07, while the ball-milled powders have average grain sizes of 10.70±2.79 and 6.64 ±1.12, respectively. The inverse pole figure (IPF) is utilized to showcase the distribution of grains within a specific area. When discussing the larger grains being located away from the patching site, it indicates that their distribution in that area is significantly distant from where the processing occurred, implying that the 150rpm does not affect the ball milling. This cluster of smaller grains may arise due to the solidification effects during the powder atomization process, though this phenomenon is not always observed. Misorientation (MO) analysis can be employed to assess material

deformation, as the relative angle between adjacent grains serves as an indicator of stress. Notably, there is no increase in MO between the untreated powder and ball-milled powders, with the untreated powder naturally exhibiting a higher intensity in MO.

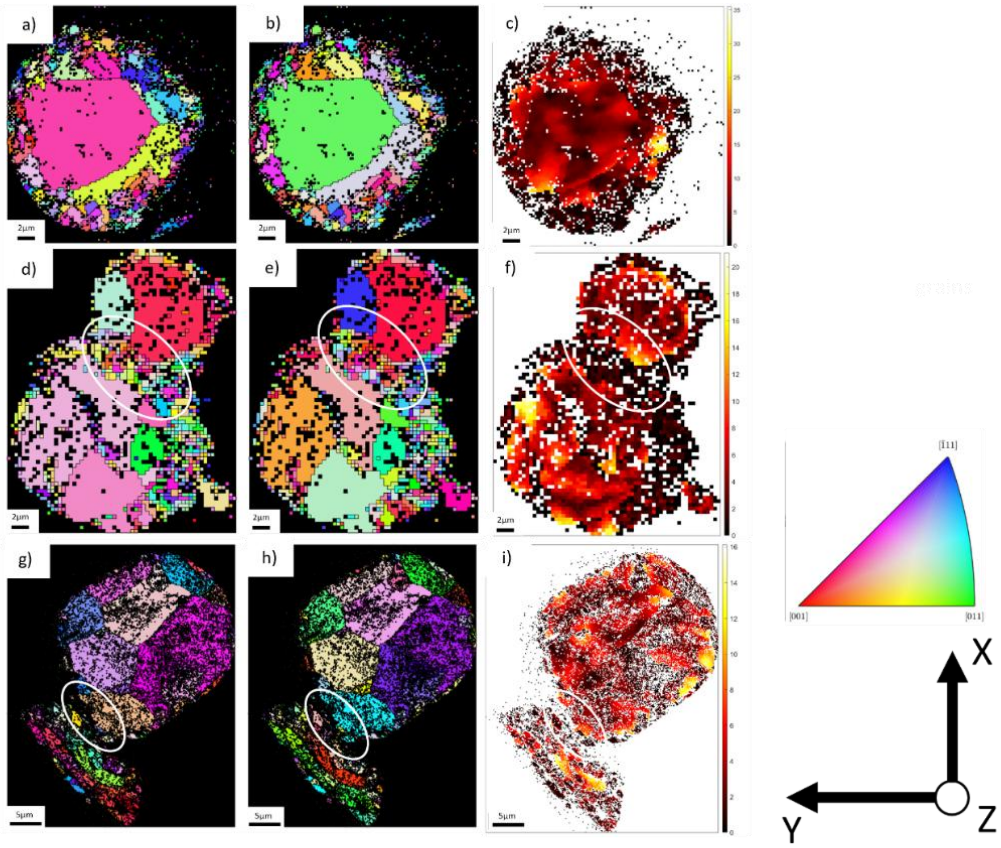

Supplementary Figure S3. EBSD IPF-X, IPF-Y, and misorientation maps of **a-c** pristine copper powder and **d-h** ball-milled copper powder, highlighting interaction zones (white circles).

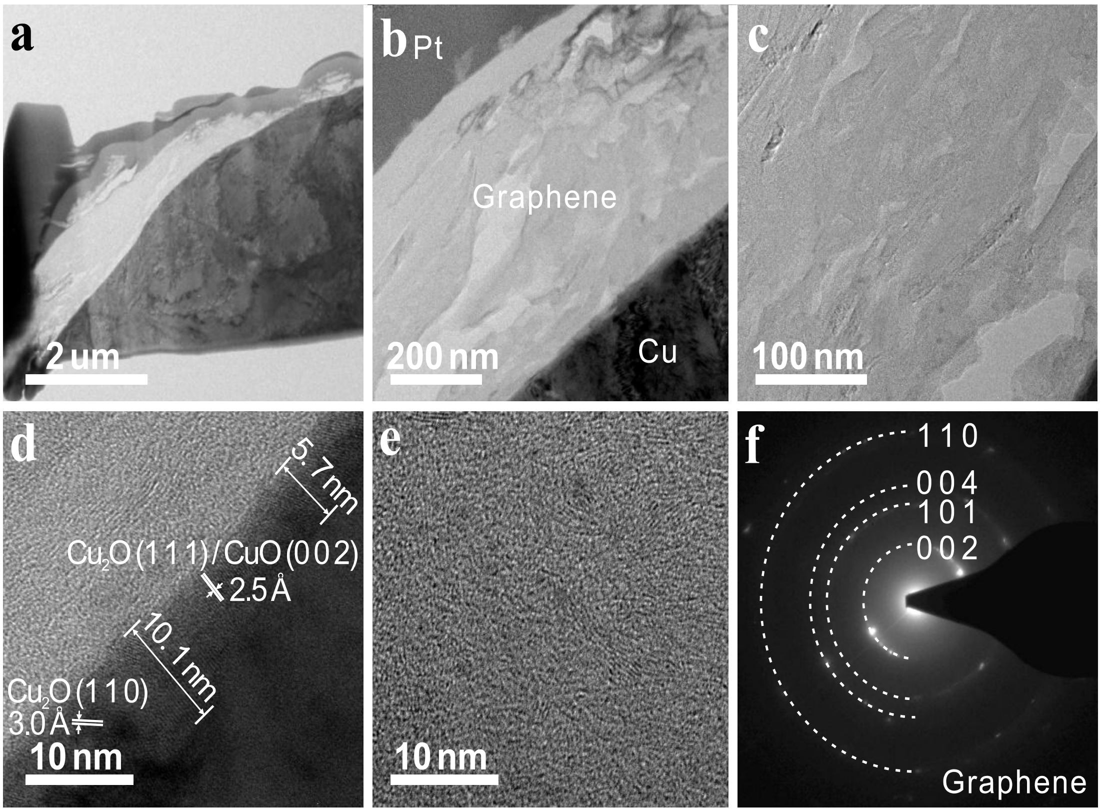

Supplementary Figure S4. Cross-sectional TEM characterization of Graphene/Cu **2D**MMC composite: **a-c** TEM images, **d-e** HRTEM images, **f** SAED pattern.

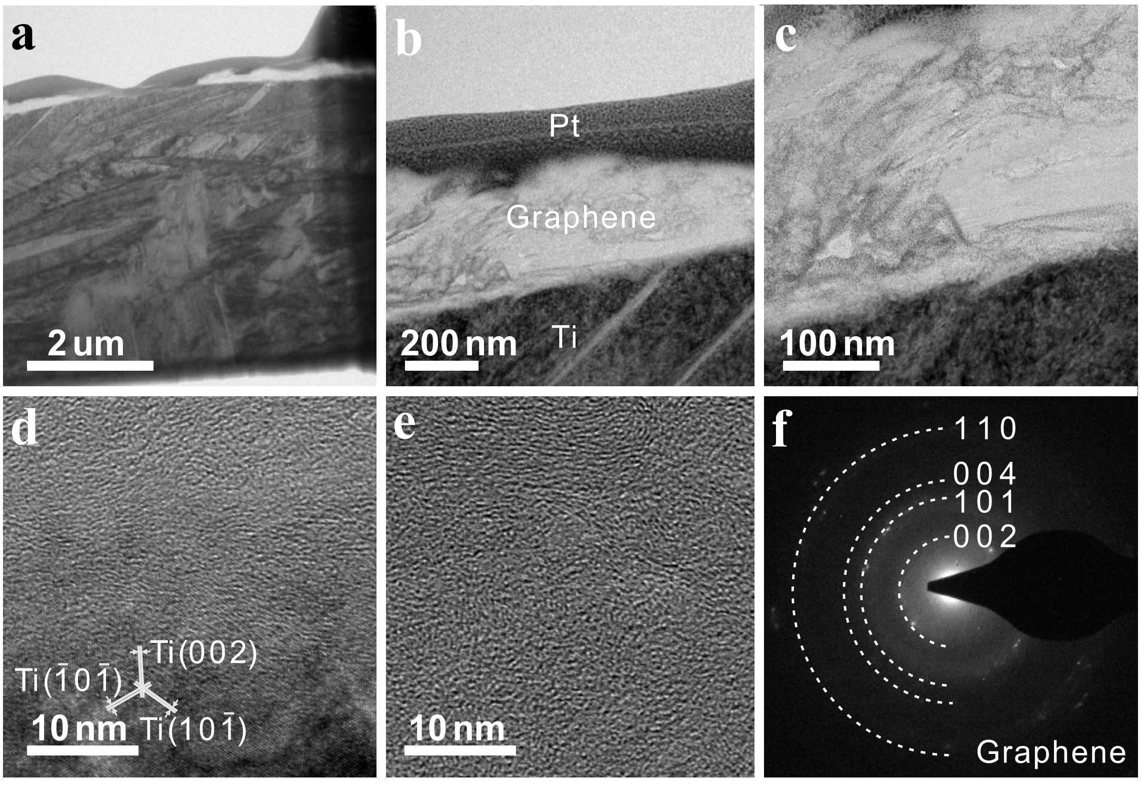

Supplementary Figure S5. Cross-sectional TEM characterization of Graphene/Ti alloy 2DMMC composite **a-c** TEM images, **d-e** HRTEM images, **f** SAED pattern.

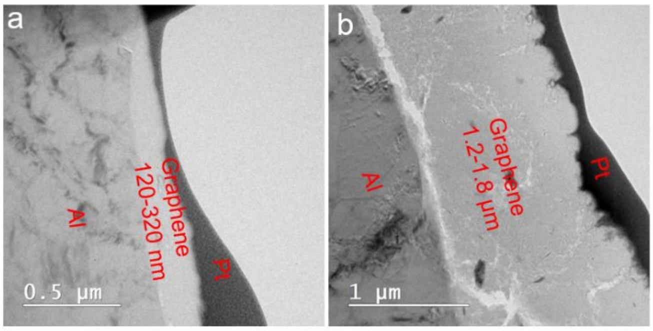

Supplementary Figure S6. Tunable graphene nanoplatelets coating on aluminum particles 2DMMCs. Low-magnification TEM images of **a** thin graphene nanoplatelet coating (~120–320 nm) aluminum particles and of **b** thick graphene nanoplatelet coating (~1.2–1.8 um) aluminum particles.

Scalability and thermal performance of 2DMMCs

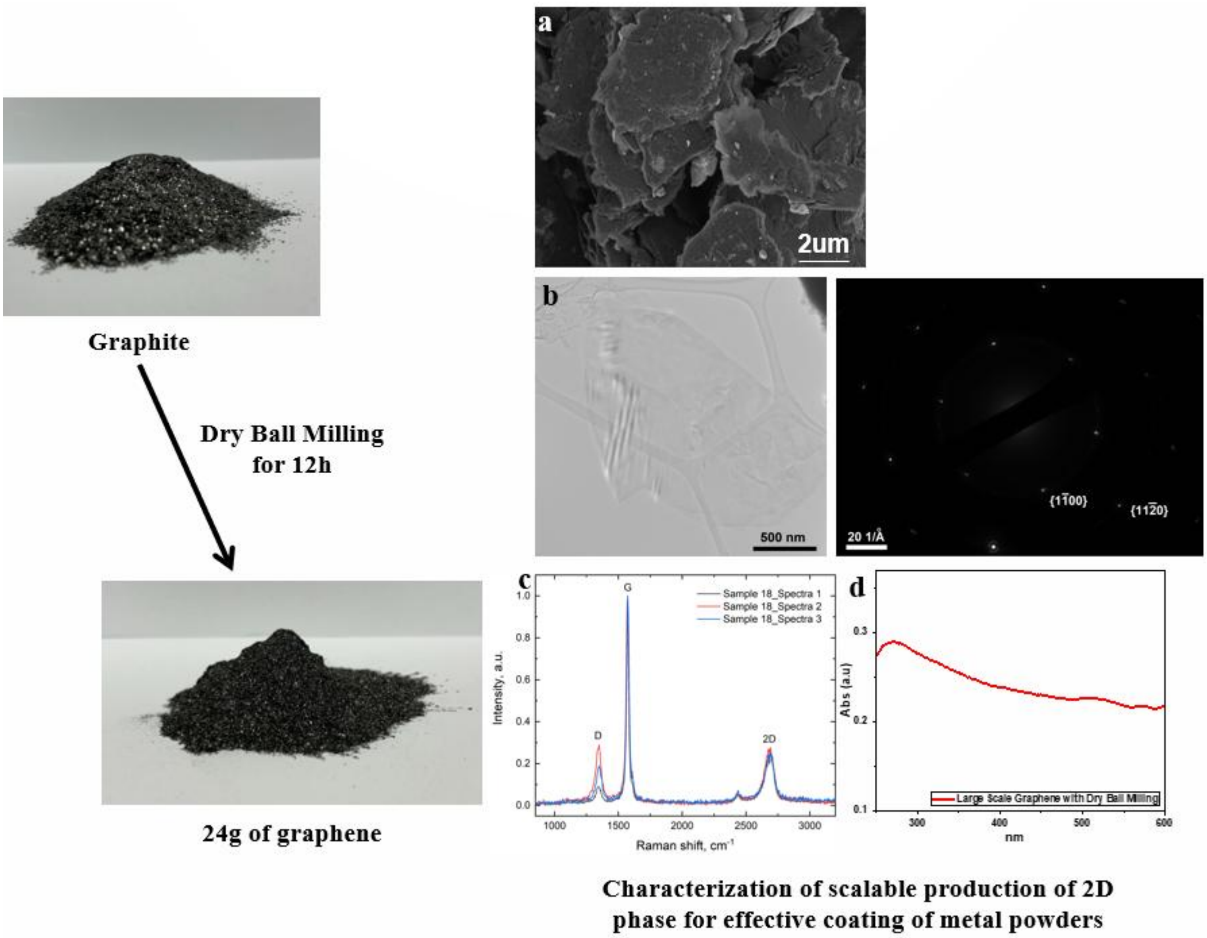

Supplementary Figure S7. Scalable production of graphene nanoplatelets (up to 48 g in 24h): **a** SEM images of the produced graphene, **b** TEM images with SAED patterns, **c** Raman spectra, **d** UV-Vis absorption spectra.

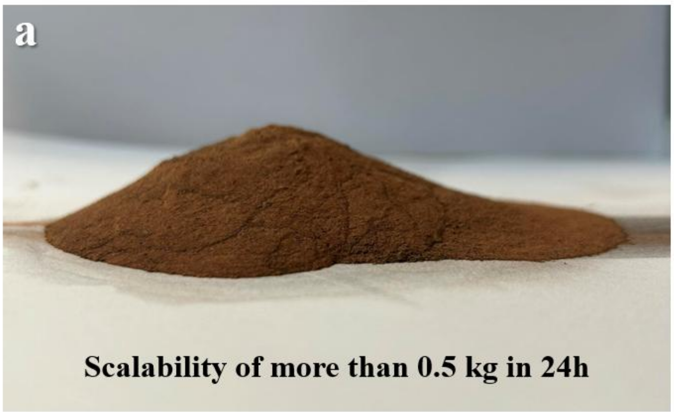

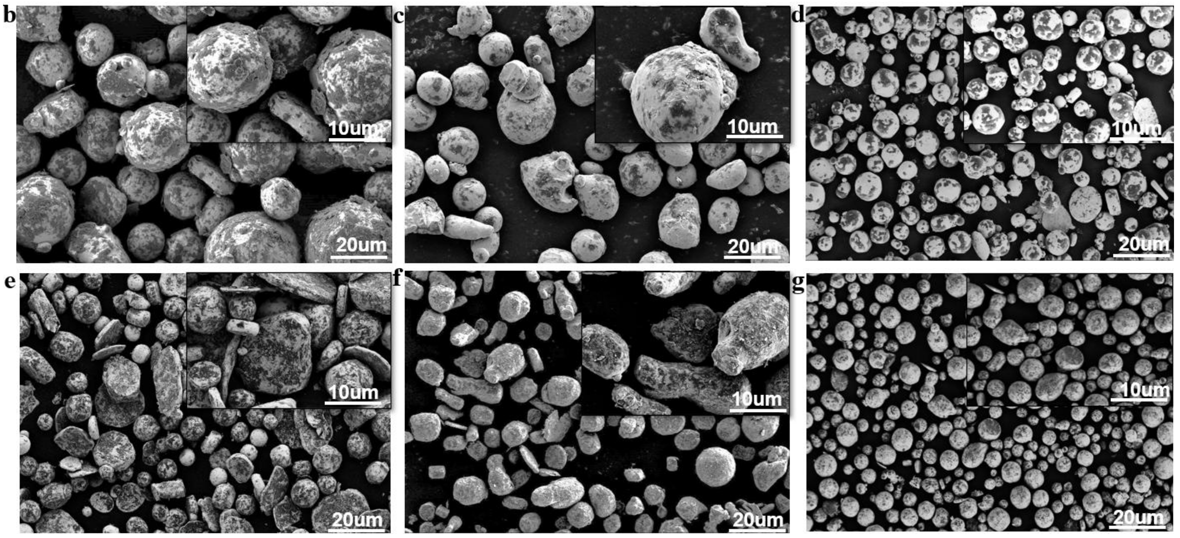

Supplementary Figure S8. a Photograph demonstrating scalable solvent-free production of graphene/Cu **2D**MMCs (>0.5 kg in 24 h). **b–g** SEM micrographs of representative 2D coated metal powders (**2D**MMCs platform) including **b** Graphene/Al alloy, **c** Graphene/Ti alloy, **d** Graphene/316L stainless steel, **e** hBN/Cu, **f** hBN/Al alloy, and **g** hBN/Ti alloy, highlighting uniform coating quality, particle morphology preservation, and scalability of the ThermoDust process.

Thermal Properties of Graphene/Ti alloy 2DMMCs

	**Thermal Conductivity (W m^{-1} K^{-1})**	**Density (g cm^{-3})**
Ti-6Al-4V	6.65 ± 0.02 (6.77)	4.4228 ± 0.0151
Solvent Free BM Ti-6Al-4V	6.60 ± 0.05 (6.47)	4.3845 ±0.039
Graphene/Ti-6Al-4V 1 wt%	6.87 ± 0.10 (6.45)	4.414 ± 0.0161
Graphene/Ti-6Al-4V 3 wt%	8.01 ± 0.14 (7.72)	4.4107 ± 0.0542
Graphene/Ti-6Al-4V 10wt%	17.33 ± 0.42 (17)	4.0644 ± 0.0097

Supplementary Table S2. Thermal conductivity of Graphene/Ti alloy **2D**MMCs and their densities. Thermal conductivity of SPS-consolidated Graphene/Ti alloy **2D**MMCs as a function of graphene nanoplatelets and the density of the final SPS composites.

**Materials**	**Thermal conductivity of matrix (W m^{-1} K^{-1})**	**Thermal conductivity of**	**Enhancement (%)**	**Reference**	**Notes**

		**composite ($W\ m^{-1}\ K^{-1}$)**			
**Ours**	**6.6**	17.3 (10wt.%)	**157 %**	-	Solvent Free Ball Milling of **2D**MMCs. Preparation of **2D**MMCs library. Scalable powder-based route with conformal coating and bulk thermal network formation.
Ti64-graphene	6.54	9.57 (10.61 vol.%)	46.3%	1 Yan, Qi, et al. Simultaneously improving mechanical, thermal, and anti-wear properties of Ti alloys using 3D-networked graphene as reinforcement. Carbon 213 (2023): 118152.	Powder metallurgy of commercial Ti64 with high graphene nanosheet loading. Quasi-continuous graphene network with in-situ TiC formation..
Ti64-*diamond*	8.3	10.4 (14 wt.%)	25.3%	2 Chen, Ling, et al. Additive manufacturing of titanium alloy-based composites using directed energy deposition: Study of microstructure, mechanical properties and thermal conductivity. *Materials Science and Engineering: A* 884 (2023): 145579.	Directed energy deposition of Ti64–diamond composites. Additive manufacturing route with moderate thermal enhancement.
Ti-graphene	14.8	15.8 (0.3wt.%)	-6.8%	3 Gürbüz, Mevlüt, Tuğba Mutuk, and Pınar Uyan. "Mechanical, wear and thermal behaviors of graphene reinforced titanium composites." *Metals and Materials International* 27.4 (2021): 744-752	Lab-scale powder metallurgy of Ti with low graphene content. Improved mechanical and thermal properties at low loading.
Ti-graphene	15.2	14.8 (0.4wt.%)	-2.7%	4 Yang, Wen-Zhi, et al. "Thermal and mechanical properties of graphene–titanium composites synthesized by microwave sintering." Acta Metallurgica Sinica (English Letters) 29.8 (2016): 707-713.	Microwave sintering of Ti–graphene composites. Lab-scale consolidation with limited scalability.
Ti-graphene	21	40 (35 vol.%)	90.4%	5 Zheng, H., & Jaganandham, K. Thermal conductivity and interface thermal conductance in composites of titanium with graphene platelets. Journal of heat transfer, (2014) 136(6), 061301	Microwave-sintered Ti–graphene composites. High conductivity at very high graphene volume fraction. Limited scalability.
Ti-graphene	22	19.3 (0.5 wt.%)	-11 %	6 Pan, Hengpei, et al. "Microstructure, mechanical and thermal properties of titanium matrix composites with different reinforcements." *Materials Research Express* 9.9 (2022): 096512	PVD-sputtered Ti–graphene thin films. Thin-film system, not bulk composite.
Ti-rGO	25.6	29.9 (6 – 10 μm thick)	16.7 %	7 Wang, Jing, et al. "Electrochemical depositing rGO-Ti-rGO heterogeneous substrates with higher thermal conductivity and heat transfer performance compared to pure Ti." Nanotechnology 28.7 (2017): 075703	Electrochemically deposited rGO–Ti layered structures. Surface coating architecture with limited scalability.
Ti-rGO	18	18.2 (2.5 wt.%)	1.1 %	#8 Liu, Jingqi, et al. "Microstructure and mechanical properties of graphene oxide-reinforced titanium matrix composites synthesized by hot-pressed sintering." *Nanoscale Research Letters* 14.1 (2019): 114.	Hot-pressed Ti–rGO composites. Powder metallurgy route with in-situ TiC formation; agglomeration at high loading.

Supplementary Table S3. Comparative table of thermal conductivity for Graphene/Ti alloy 2DMMCs obtained via different processing approaches.

LPBF of Graphene/Ti Alloy and hBN/Ti Alloy 2DMMCs

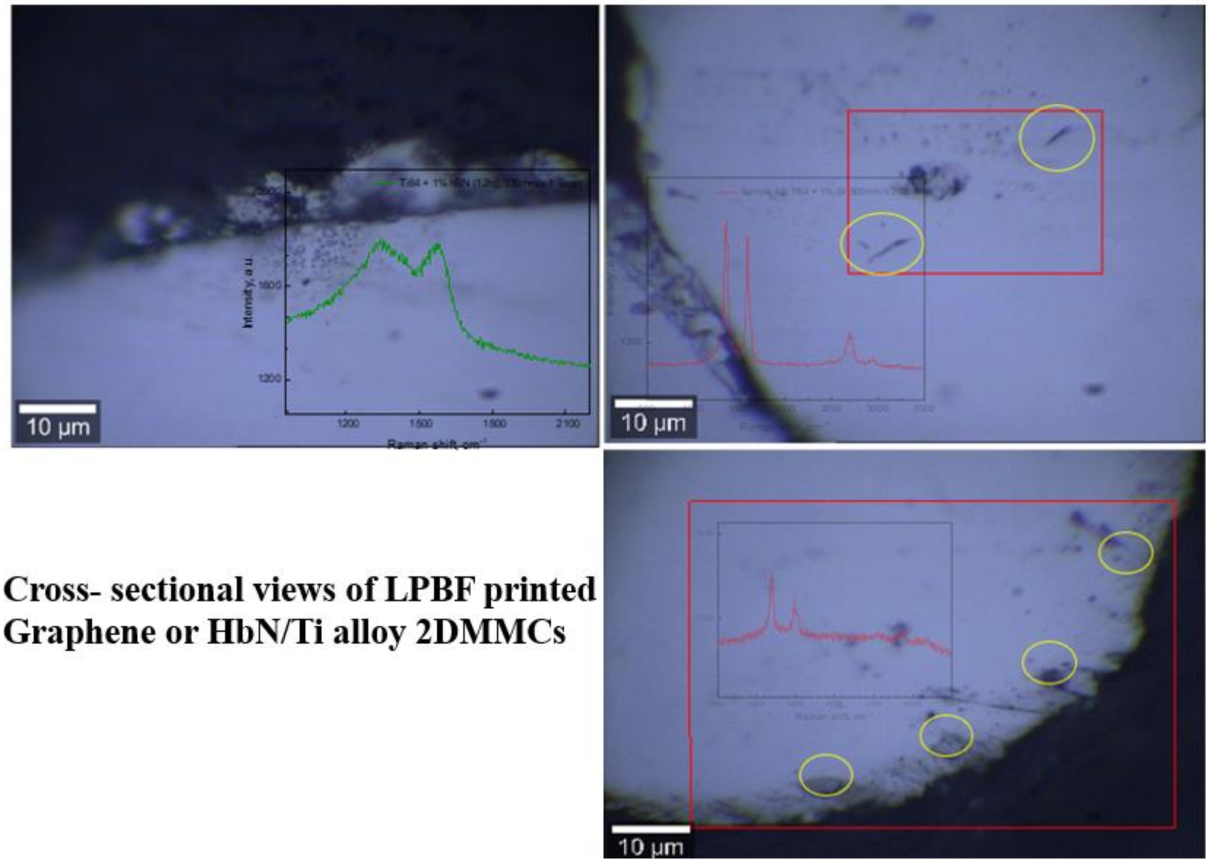

Supplementary Figure S9. Single-layer LPBF printing of graphene- and hBN-reinforced Ti alloy composites (1 wt% and 10 wt% 2D phase) and graphene-reinforced 316L stainless steel **2D**MMCs.

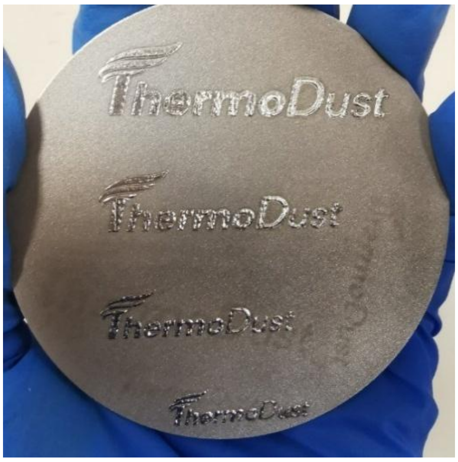

Supplementary Figure S10. LPBF-printed "ThermoDust" demonstrator fabricated from graphene/Ti alloy **2D**MMC powder.

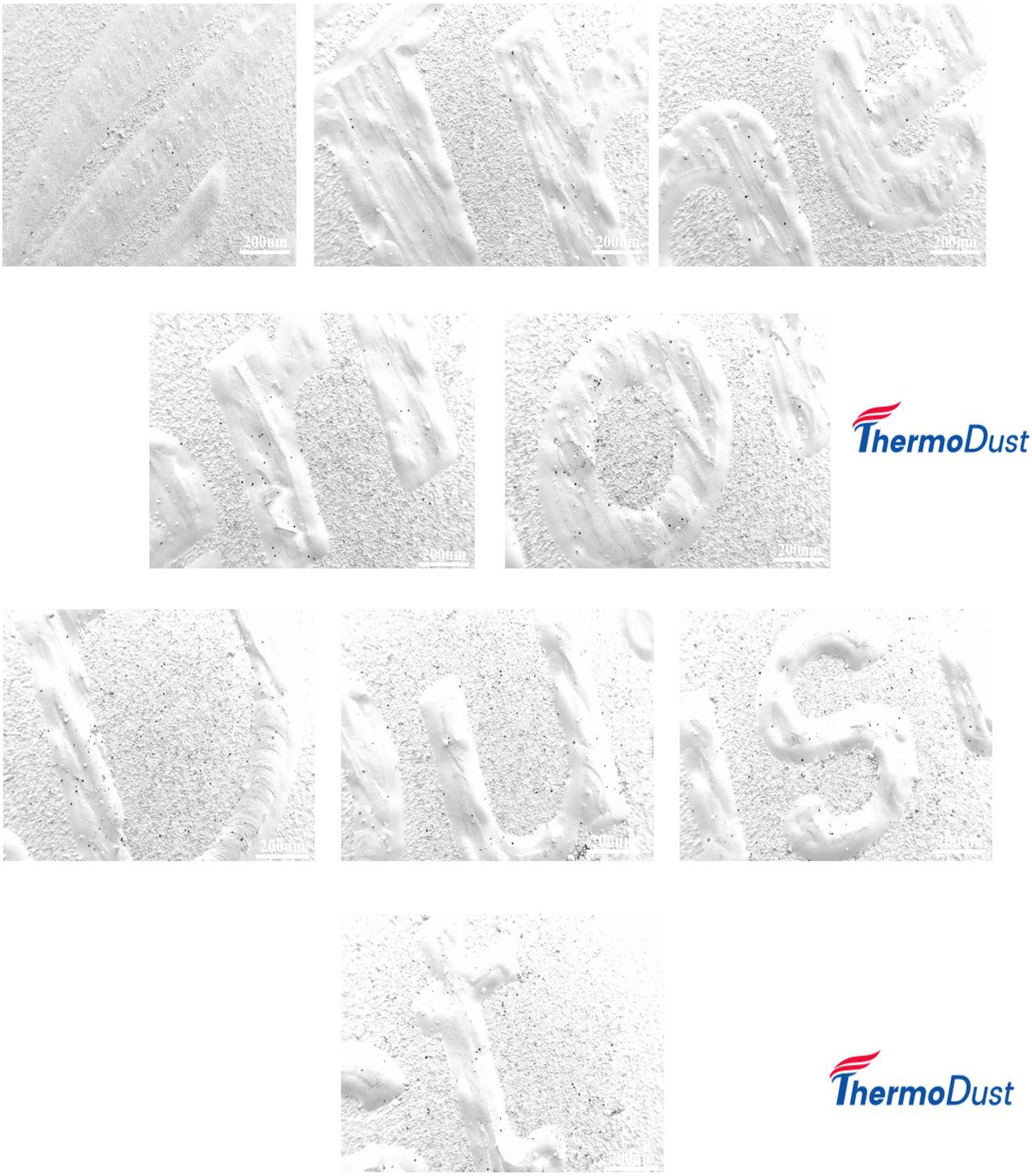

Supplementary Figure S11. SEM images of the LPBF-printed "ThermoDust" pattern (200 μm scale), highlighting the high printing resolution and accurate reproduction of the designed geometry.
